# Perianal Langerhans cell hyperplasia characterized by perianal ulcerative lesions after hemorrhoid surgery: A case report

**DOI:** 10.1097/MD.0000000000046381

**Published:** 2025-12-12

**Authors:** Dexue Hua, Yahui Han, Dan Huang, Wenbo Zheng, Lingmin Zheng, Jitao Fu, Shubo Gao

**Affiliations:** a Department of Anorectal Surgery, Affiliated Hospital of Jining Medical University, Jining China.

**Keywords:** case report, haemorrhoid, Langerhans cell hyperplasis, perianal, skin ulcerative

## Abstract

**Rationale:**

Langerhans cell histiocytosis (LCH) is a rare disorder with diverse, nonspecific manifestations, posing significant diagnostic challenges. Perianal involvement is exceptionally uncommon and can be easily misdiagnosed as common benign conditions, especially following anorectal surgery.

**Patient concerns:**

A 76-year-old male with a history of chronic obstructive pulmonary disease and coronary artery disease presented with refractory perianal ulcerative lesions that developed after a hemorrhoid surgery. The patient’s primary concern was nonhealing, painful perianal ulcers despite initial standard wound care.

**Diagnoses:**

Histopathological and immunohistochemical analysis of the debrided tissue confirmed the diagnosis of LCH (CD1a+, CD207+). A subsequent systemic positron emission tomography/computed tomography scan revealed multifocal LCH involvement in the ileocecal region, bones, and other sites, confirming multisystem disease.

**Interventions:**

The patient underwent local surgical debridement and wound care. Upon diagnosis of multisystem LCH, he was referred to hematology and received systemic chemotherapy with cytarabine.

**Outcomes:**

Following chemotherapy, the perianal ulcer was nearly healed. However, due to poor tolerance and severe gastrointestinal side effects, the patient voluntarily discontinued treatment. This led to a recurrence of the ulcer and a tendency toward anal stenosis.

**Lessons:**

This case highlights that perianal LCH can masquerade as a nonhealing surgical wound. Its diagnosis requires a high index of suspicion and timely biopsy with immunohistochemical confirmation. Early and accurate diagnosis is crucial for initiating appropriate systemic evaluation and individualized management, which must carefully balance treatment efficacy with patient tolerance to prevent the morbidity associated with progressive multisystem disease.

## 1. Introduction

In clinical practice, Langerhans cell histiocytosis (LCH) is relatively rare, with various clinical manifestations and lack of specificity. Clinicians lack systematic understanding of LCH, which is likely to lead to missed diagnosis, misdiagnosis and mistreatment. LCH occurring in perianal skin is even rarer. Here, the diagnosis and treatment process of a patient with perianal LCH in our hospital is shared as follows and relevant literature is reviewed, in order to provide reference for the diagnosis and treatment of perianal LCH.

## 2. Case presentation

This article reports a 76-year-old patient who underwent surgery for hemorrhoids. The patient had poor postoperative wound healing and evolved into ulcerative lesions, so the second surgical treatment was performed, and the pathological diagnosis was Langerhans cell hyperplasia with multisystem lesions. In the later stage, the patient received cytarabine chemotherapy and local dressing change, and the ulcer was close to healing. Due to the poor tolerance of chemotherapy, the patient discontinued chemotherapy treatment, which led to the recurrence of ulcers.

## 3. Case description

**Figure 1.**
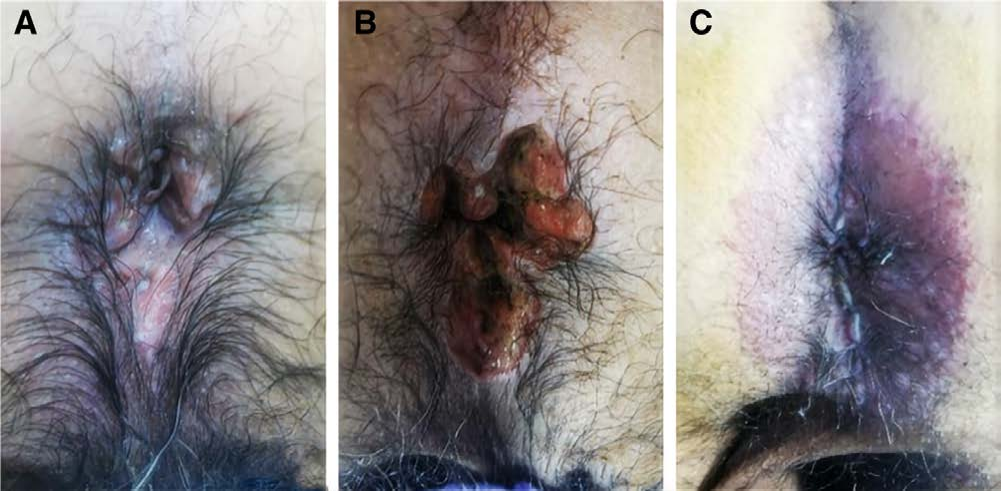
Preoperative perianal skin lesions and postoperative wound surface. (A) Before the operation, the anus had an uneven appearance, with ulcers approximately 1.5 cm in diameter and moss-like changes visible on the right and left rear sides. (B) 6 days after the operation, multiple surgical wounds were visible around the anus, with fresh granulation tissue, local inflammatory secretions, and an acceptable surrounding skin color. (C) 4 mo after the operation, the skin within a radius of approximately 3 cm outside the anus was purple–red, rough, and moss-like, with scattered papules and local exudation.

The patient is an elderly 76-year-old male with a history of chronic obstructive pulmonary disease and coronary artery disease who underwent surgery for mixed hemorrhoids in a local hospital in June 2021, and the specimen was not sent for pathology. During the postoperative recovery process, the skin around the incision is repeatedly flushed, broken, ulcerated, and painful. The patient’s symptoms did not improve significantly after the use of dressing change, traditional Chinese medicine sitz bath, and a variety of ointments for external treatment. So the patient came to our department for treatment. The patient was preliminarily diagnosed with “poor healing of postoperative anal wounds” and was hospitalized. At the time of admission, the patient experienced perianal pain from time to time, but his general condition was fine, his mental state was clear, his spirit was fine, his appetite and sleep were fine, his urination and defecation were normal, and his body weight did not change significantly. Specialized examination revealed that the anus had an uneven appearance, with an ulcerated surface approximately 1.5 cm in diameter and mossy lesions on the right posterior and left posterior sides (Fig. 1A), with obvious tenderness, no obvious abnormality in the lower part of the anorectal canal on palpation, and no blood staining on the finger cuffs.

**Figure 2.**
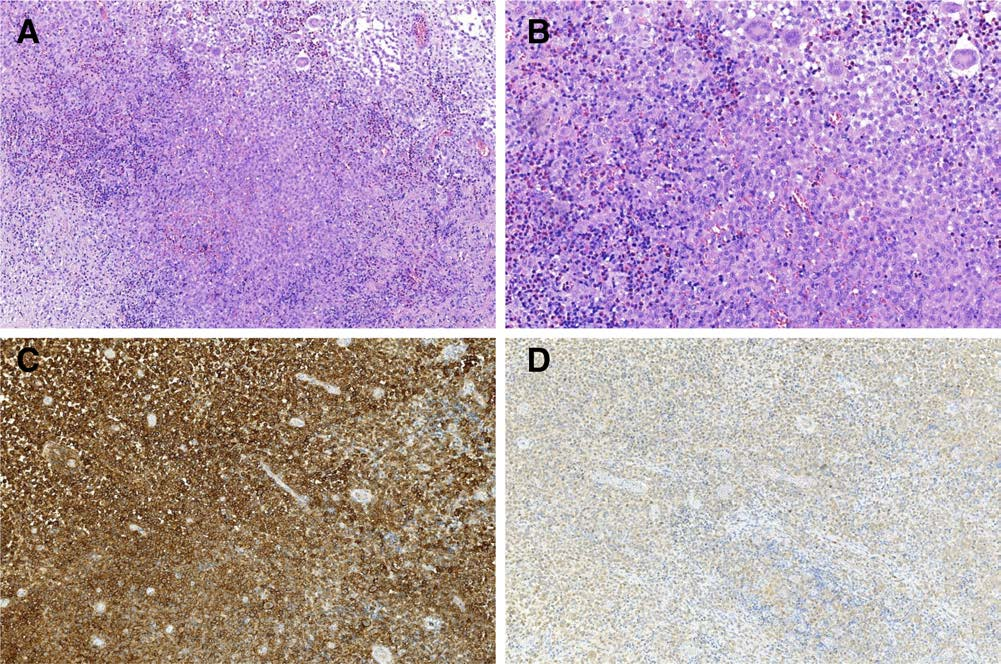
Histopathological and immunopathological images of perianal Langerhans cell hyperplasia. (A) LCH cells were dispersed in the field of visual field. Multinucleated giant cells and eosinophils were seen in the background. (B) Typical Birbeck particles were observed. Nuclei are irregular, with nuclear furrows, curled and folded. (C) The results of immunohistochemistry showed that the expression of CD1a was positive, which was mainly distributed in the cell membrane. (D) The expression of CD207 was positive, mainly distributed in the cell membrane and cytoplasm, and granular cytoplasm was seen. (A: HE × 100; (B) HE × 200; (C) CD1a × 100; (D) CD207 × 100).

The major laboratory test results were as follows: blood tests revealed leukocytes 5.73 × 10^9^/L, neutrophils 3.72 × 10^9^/L, a neutrophil percentage of 65%, and lymphocytes 1.44 × 10^9^/L; carcinoembryonic antigen (CEA), alpha-fetoprotein (AFP), carbohydrate antigen (CA) 50, CA242, CA19-9, CA125, neuron-specific enolase, and squamous cell carcinoma antigen (SCC) levels were all normal; and liver and kidney function, electrolytes, and coagulation function tests revealed no obvious abnormalities in liver or kidney function, electrolytes or coagulation function. The patient’s admission diagnosis was poor healing of the incision after hemorrhoid surgery. After contraindications to surgery were excluded, perianal skin and subcutaneous tissue debridement was performed, under moderate sedation and local infiltration anesthesia. Postoperatively, ceftriaxone sodium injection was given to prevent infection, and the incision was treated with daily dressing changes. The patient’s 6-day postoperative wound condition is shown in Figure 1B. Postoperative pathology revealed (Fig. 2): localized breakage of the skin epidermis of the perianal canal, a large number of histiocyte aggregates, a multinucleated giant cell reaction and eosinophilic granulocyte infiltration in the skin. These findings, combined with the results of immunohistochemical labeling, were consistent with LCH. The immunohistochemistry results were as follows: cluster of differentiation (CD)68 (+), CD1a (+), terminal deoxynucleotidyl transferase (TdT) (-), CD207 (+), and nuclear proliferation antigen (Ki-67) (+, localized approximately 30%). Therefore, the diagnosis was corrected to perianal LCH. The patient was asked about his medical history: he was treated with surgery in a provincial hospital 7 years prior for a left parotid cyst, and the postoperative pathology suggested LCH, but he was not further diagnosed and treated at that time.

The patient underwent wound dressing changes for 4 months, but the effect was not good. There was still an ulcer in the perianal area with painful exudation (Fig. 1C), so he went to the hematology department for chemotherapy. The patient completed the positron emission tomography/computed tomography (PET/CT) examination: There is a high possibility that the anal area will be infiltrated by LCH. The ileocecal region, external auditory canal, ureter, seminal vesicle glands, ribs and acetabulum are likely to be infiltrated by LCH. Diffuse mild hypermetabolism of bone throughout the body. The left parotid gland was surgically removed. Bone marrow puncture revealed good bone marrow granulocyte hyperplasia, a reduced proportion of erythroid cells, and an increased proportion of monocytes. No obvious morphological abnormalities were found. The hematology department provided multiple rounds of cytarabine regimen chemotherapy and dressing changes, and the ulcer was close to healing. However, owing to the patient’s poor tolerance to chemotherapy and severe gastrointestinal reaction(diarrhea, vomiting, nausea, abdominal pain), the treatment was interrupted on his own, resulting in recurrence of the ulcer and a tendency for anal stenosis.

## 4. Discussion

LCH is a rare hematopoietic neoplasm originating from myeloid dendritic precursor cells,^[1]^ a group of lesional entities characterized by Langerhans cell proliferation.^[2]^ It has several features of both inflammatory reactivity and clonal growth.

LCH develops in all age groups, but the age of onset is generally relatively young. In terms of sex, the incidence rate is significantly higher in males than in females.^[3]^ Critically, its clinical manifestations are diverse and strikingly lack specificity, which constitutes a major barrier to timely recognition and frequently leads to delayed diagnosis, particularly in anatomically complex or surgically altered sites like the perianal region. The degree of disease involvement can be divided into mild single-system lesions with self-repair and multisystem lesions that are severely impaired or even life-threatening.^[4,5]^ This case vividly illustrates these diagnostic hurdles. The patient presented solely with perianal symptoms initially attributed to hemorrhoids. Following debridement of the diseased perianal skin, pathological examination surprisingly revealed LCH. Prompted by this unexpected finding and underscoring the systemic nature of multisystem LCH, we performed PET/CT examination, which found LCH infiltrates in many tissues throughout the body. Bone marrow aspirate ruled out myelopathy. Thus, the initial localized presentation masked a systemic disease, highlighting the imperative for comprehensive investigation following an LCH diagnosis in any location, especially given its potential for multisystem involvement.

When LCH invades the skin system, it may appear as a scaly, oily rash or small erythematous papules, as well as red or purple nodules, ulcers, and abscesses, and the skin system may be the only manifestation of LCH.^[6,7]^ However, LCH involving the perianal skin isexceedingly less common. Through a review of the literature, we found that LCH can be a clinical symptom of “perianal ulcer, inflammatory response, pruritus” in the perianal aream.^[8]^ In this case, the initial misdiagnosis of “hemorrhoids” underscores the profound diagnostic challenge. The perianal manifestations – an uneven appearance, redness, mossy changes, and ulcers on the right and left posterior sides with obvious tenderness – were consistent with but not pathognomonic for LCH. This nonspecific presentation readily mimics benign conditions, delaying suspicion of a neoplastic process like LCH. Consequently, in clinical practice, perianal LCH demands careful differentiation from a wide array of conditions, including flat warts, condyloma acuminatum, soft chancres, Crohn disease, cutaneous tuberculosis, mycobacterial infections, deep-seated fungal diseases, and importantly, malignancies such as squamous cell carcinoma or anal melanoma.^[9]^ Given that the treatment of LCH is radically different from that of benign perianal disease or other malignancies, a high index of suspicion is paramount. Biopsy of suspicious perianal lesions remains the indispensable cornerstone for accurate diagnosis and appropriate management within an oncology setting.

Histopathologic and immunohistochemical examinations are the main basis for the diagnosis of LCH. Microscopically, LCH cells have irregular nuclei with obvious folds and grooves, fine chromatin and blurred nucleoli. The tissue is infiltrated with varying degrees of eosinophils, lymphocytes, histiocytes, and neutrophils. The immunophenotypic features of LCH include the expression of CD1a, the S100 protein, and Langerin (CD207). Birbeck granules can be found in the cytoplasm via electron microscopy. The presence of Birbeck granules or CD1a antigen positivity is the gold standard for the diagnosis of LCH.^[10]^ Another study revealed that a significant proportion of LCH patients have BRAF V600E gene mutations, and BRAF V600E measurement of pathologic tissues is helpful for diagnosis and differential diagnosis.^[11]^

Due to the rarity of the disease and incomplete understanding of its pathophysiological properties, the current treatment of LCH is not standardized. Hamdan M reviewed the care of 15 patients with perianal LCH and found that patients were initially treated with antibiotics and corticosteroids, and if the lesion was refractory, systemic chemotherapy was started. Only 3 of these patients underwent surgical intervention[8]. In our patient, diagnosed with multisystem LCH, systemic chemotherapy with cytarabine was initiated. Our regimen is similar to that reported by Mansour MJ et al,^[12]^ whose patients underwent a 29-cycle chemotherapy regimen of cytarabine plus vinblastine. While Mansour et al reported good tolerance and significant reduction in colorectal involvement, our patient experienced severe gastrointestinal reactions (vomiting, abdominal pain, diarrhea) leading to treatment interruption. This disparity in tolerance underscores the critical need for individualized treatment strategies in oncology practice, particularly considering patient factors like age and comorbidities. Our patient was older with potentially poorer underlying fitness, contributing to the intolerance. Consequently, compliance decreased, and the opportunity to trial alternative regimens was lost. Persistent perianal ulcerative lesions and a tendency towards anal stenosis were observed during follow-up, illustrating the suboptimal disease control achieved and the challenges in managing complex perianal manifestations of LCH.

This experience emphasizes that treatment must be individualized based on the extent of disease involvement and patient factors. For patients with single-system LCH, options include clinical observation, resection, or localized therapy, with vigilant monitoring for progression.^[13,14]^ However, patients with multisystem involvement, like ours, require prompt systemic therapy. For perianal LCH involving multiple systems, there is currently no unified chemotherapy regimen. Our case demonstrates that when a single regimen is poorly tolerated or response is suboptimal, sequential or combination regimens may be necessary. Furthermore, given the protracted nature of LCH and the specific morbidity of perianal lesions, the importance of integrating local therapies (e.g., specialized wound care, topical agents, potentially targeted therapies based on mutational status) alongside systemic treatment cannot be overstated. A multimodal approach combining, for example, perianal wound management (dressing changes), adjunctive therapies (e.g., herbal fumigation, if evidence-based), and tailored systemic therapy holds promise for improving outcomes in these complex cases.

However, the patient underwent resection of a cystic lesion in the parotid gland 7 years ago. The histopathological report at that time was suggestive of LCH, but a definitive diagnosis could not be established since immunohistochemistry was not performed on the specimen. The current anorectal involvement may represent a secondary manifestation resulting from the lack of appropriate treatment at the initial presentation. To clearly distinguish between Langerhans cell hyperplasia and LCH, additional studies – such as cyclin D1 immunostaining and mitogen-activated protein kinase/v-Raf murine sarcoma viral oncogene homolog B (BRAF) mutation analysis – are necessary.

In conclusion, this case powerfully illustrates the significant diagnostic difficulty inherent in perianal LCH, particularly following common anorectal surgery. The lack of specific symptoms, the mimicry of benign conditions or other pathologies (including malignancy), and the sheer rarity in this location contribute to a high risk of delayed or missed diagnosis. It is imperative for clinicians managing perianal disorders, especially colorectal surgeons and oncologists, to include LCH in the differential diagnosis of refractory or atypical perianal ulcers, even post-hemorrhoidectomy, and to maintain a low threshold for biopsy. Histopathology with appropriate immunohistochemistry (CD1a, S100, Langerin) is essential for definitive diagnosis. This case serves as a crucial clinical alert: early recognition and biopsy of suspicious perianal lesions are vital to initiate appropriate systemic evaluation and timely, individualized oncologic management, thereby preventing the significant morbidity associated with delayed treatment in multisystem LCH.

## 5. Conclusions

The perianal manifestations of Langerhans cell hyperplasia are complex and varied, and early diagnosis is critical for the treatment of the disease.

## Acknowledgments

We thank the patient for permitting us to use his data to complete this article.

## Author contributions

**Study design and direction of the project:** Shubo Gao.

**Collection of data:** Dexue Hua, Yahui Han, Lingmin Zheng, Dan Huang.

**Analysis and writing – original draft:** Jitao Fu, Shubo Gao.
